# In-hospital Mortality and Causes of Death in People Diagnosed With HIV in a General Hospital in Shenyang, China: A Cross-Sectional Study

**DOI:** 10.3389/fpubh.2021.774614

**Published:** 2021-11-30

**Authors:** Cheng Bo Li, Ying Zhou, Yu Wang, Sheng Liu, Wen Wang, Xu Lu, Cui Ming Sun, Pei Liu, Qing-Hai Hu, Ying Wen

**Affiliations:** ^1^Infectious Diseases Department, The First Affiliated Hospital of China Medical University, Shenyang, China; ^2^Key Laboratory of AIDS Immunology of Ministry of Health, Department of Laboratory Medicine, The First Affiliated Hospital of China Medical University, Shenyang, China

**Keywords:** acquired immune deficiency syndrome, in-hospital mortality, antiretroviral therapy, intensified medical care strategy, cross-sectional study

## Abstract

**Background:** Acquired immune deficiency syndrome (AIDS), caused by human immunodeficiency virus (HIV) infection, is a serious public health issue. This study investigated the correlated factors and possible changing trend of in-hospital death in patients diagnosed with HIV in the past decade in our hospital.

**Methods:** We retrospectively collected data of firstly hospitalized patients with HIV in the Department of Infectious Disease in the First Affiliated Hospital of China Medical University from January 1, 2010 to December 31, 2019, and compared various factors that correlated with in-hospital death, including age, sex, opportunistic infections, and antiretroviral therapy (ART) status. Cox regression analysis was used to identify the risk factors for death.

**Results:** In total, 711 patients were recruited for this study, and 62 patients died in the hospital. The in-hospital mortality rate was 8.72%. Tuberculosis (TB), malignancies, and thrombocytopenia were associated with mortality. Antiviral treatment before admission was found to be a protective factor. There was a declining trend in in-hospital mortality from 19.2% in 2010 to 6.3% in 2019 (linear-by-linear association test, *p* < 0.001), partly due to intensified medical care strategy.

**Conclusions:** Till date, AIDS-defining illnesses remain the major cause of hospital admission and in-hospital mortality. TB and malignancies were correlated risk factors for in-hospital mortality. ART before admission was found to be beneficial, and considering the decreasing rate of in-hospital mortality, the implementation of intensified medical care strategy requires further effort.

## Introduction

Acquired immune deficiency syndrome (AIDS), caused by human immunodeficiency virus (HIV) infection, is a serious public health problem, being the sixth most common cause of mortality worldwide (1). CD4+T lymphocytes destruction leads to severe immune deficiency, which causes various opportunistic infections and malignant tumors. Late diagnosis and late treatment lead to high morbidity and mortality rates. With the employment of effective antiretroviral therapy (ART) and an updated strategy of ART initiation, AIDS-defining deaths have reduced significantly. Non-AIDS-related mortality rate is increasing among well-virologically controlled populations living with HIV, especially in persons with previous AIDS-defining events (2, 3). However, the death spectrum of inpatients might be different from that of enrolled outpatients undergoing ART in a long-term follow-up cohort study.

Since June 15, 2016, the time to initiate ART has been updated based on CD4+T cell levels in China, as AIDS-related illnesses remain the leading causes of hospital admission and mortality worldwide (4, 5). Risk factors for mortality should be surveyed in a timely manner and effectively addressed. Patients with HIV-related or ART-related complications are more likely to be admitted to the infectious disease department, while patients with age-related comorbidities are more likely to be initially admitted to other departments in the general hospital. In resource-limited hospitals, laboratory capabilities and medical team standards are limited. Therefore, enhanced communication and cooperation between infectious disease specialists and other healthcare specialists are required.

For the past few years in our hospital, implementing regular screening for comorbidities in hospitalized HIV patients, training of an HIV specialist team, and constructing a multidisciplinary team (MDT) have demonstrated diagnostic and therapeutic advantages. This study is a retrospective analysis of the correlated risk factors and the possible change in trend for in-hospital mortality in the past decade.

## Materials and Methods

### Patients

We conducted a retrospective study of inpatients who had been diagnosed with HIV (infection) and were initially admitted to the Department of Infectious Disease at the First Affiliated Hospital of China Medical University (Shenyang, China) from January 1, 2010 to December 31, 2019. Patients with incomplete data were excluded from the study. This study was approved by the Clinical Research Ethics Committee of China Medical University. Our hospital is a tertiary hospital with 3933 beds. For the HIV care in the Department of Infectious Disease, we serve out-patients (at least 4,500 patients in the follow-up) and in-patients (90 beds in the ward for various infectious diseases). HIV in-patients came from ourselves HIV care clinic and different cities around Shenyang.

### Study Design

The prognosis of patients who survived or died was recorded when they were discharged from the hospital. Clinical data, including demographic data, underlying medical conditions, and clinical presentations, were obtained from the patients' medical records, which were completed by three authors simultaneously. We separately analyzed the factors that might influence mortality rate, including age, sex, body mass index (BMI) <16 kg/m^2^, history of smoking and alcohol abuse, World Health Organization (WHO) clinical stage classification, length of hospital stay, fever duration >7 days before admission, CD4+T cell count, hemoglobin (HB) level, platelet (PLT) count, glutamic alanine transaminase (ALT) level, serum albumin (ALB) level, serum creatinine (Scr) level, serum sodium concentration, C-reactive protein(CRP) level, dyslipidemia, viral hepatitis, respiratory failure, tuberculosis (TB), pneumocystis *jiroveci* pneumonia (PJP), central nervous system (CNS) infection, malignancies, invasive fungal infection, and bacterial bloodstream infection. We also analyzed the trend of in-hospital mortality rates, CD4+T cell counts <200 cells/μL, advanced WHO stages, and ART status before admission among inpatients during the study period.

### Definitions

Patients who had been taking ART for any treatment duration time at admission were defined as ART prior to admission. Patients with HIV-1 RNA of at least 1,000 copies/mL after initiating ART for at least 6 months (ART-experienced) should undergo drug resistance testing by first-generation sequencing, with drug resistance defined as intermediate or high-level resistance using the Stanford HIV drug resistance program. Hyponatremia was defined as serum sodium concentration <135 mmol/L. Thrombocytopenia was defined as a platelet count <1,00,000/μL. Fever duration >7 days before admission was defined as the duration of fever persistence from onset to admission for more than 7 days. In this study, invasive fungal infections were defined as disseminated fungal infection or invasive pulmonary fungal infection. The WHO clinical stage for all patients was based on the most serious clinical stage in their medical history records. Intensified medical care strategies included regular screening for comorbidities and training of HIV care clinic staff and the MDT.

### Statistical Analysis

The results are expressed as median (interquartile range), numbers, and percentages. We compared the conditions between those who survived and those who died. The means for continuous variables were compared using the Student's *t*-test for normally distributed data. Otherwise, the Mann-Whitney *U*-test was used. The proportions for categorical variables were compared using the chi-squared test, and Fisher's exact test was used when the data were sparse. The Kaplan–Meier method and Cox proportional hazard regression model were employed to identify the correlated risk factors for mortality. Data with a *P* < 0.1 in the univariable analysis were entered into the multivariate Cox proportional hazard model. The hazard ratio (HR) was computed with a 95% confidence interval (CI), and *P* < 0.05 were considered statistically significant for all cases. Mortality rates, ART status before admission, advanced WHO stages, and CD4+T cell count were evaluated by the linear-by-linear association to identify possible changing trends during the past decade. All analyses were performed using SPSS software for Windows (version 22.0; Chicago, IL).

## Results

### Clinical and Laboratory Characteristics at Baseline

In total, 711 patients were enrolled in this study, including 659 men (92.7%) and 52 women (7.3%). The median age was 37 years (range: 16–79 years). HIV was sexually transmitted in 653 patients (91.8%), and 412 patients were men who have sex with men (MSM). Among nine patients with cirrhosis, six were in a decompensated cirrhosis state. Nobody met the diagnostic criteria of alcohol abuse.

In total, 192 patients were admitted after June 15, 2016. Among 143 patients (20.1%) whose cause of hospital admission was adverse drug event, 105 (73.4%) experienced this event before June 15, 2016. The causes of hospital admission in 532 cases (74.8%) were AIDS-related illnesses, in other words being at WHO clinical stage III–IV, including candidiasis, PJP,TB, CNS infection, invasive fungal disease, cytomegalovirus (CMV) retinitis, malignancies, bacterial bloodstream infection, and disseminated *Mycobacterium avium* complex disease. The common laboratory test abnormalities were summarized in Table 1.

**Table 1:** Baseline clinical and laboratory characteristics of 711 hospitalized HIV patients.

**Data**	**No. (%)**
Age (years) <30	178 (25.0%)
30–49	375 (52.7%)
≥50	158 (22.2%)
Male	659 (92.7%)
ART prior to admission	308 (43.3%)
ART <6 m	156
ART >6 m without virological failure	121
ART >6 m with virological failure	31
The interval of fever (days) >7	456 (64.1%)
BMI <16 kg/m^2^	32 (4.5%)
Hepatitis B virus-coinfection	73 (10.3%)
Hepatitis C virus-coinfection	9 (1.3%)
Hyperlipidemia	166 (23.3%)
Diabetes	30 (4.2%)
Hypertension	28 (3.9%)
The cigarette index >400	137 (19.3%)
Admission after 20160615	192 (27.0%)
The causes of hospital admission	
AIDS-related illnesses	532 (74.8%)
Adverse drug event	143 (20.1%)
Others	36 (5.1%)
WHO clinical stage III–IV	532 (74.8%)
Candidiasis	358 (50.4%)
PJP	350 (49.2%)
Respiratory failure	142
TB	282 (39.7%)
Respiratory failure	5
Extra-pulmonary TB	101
CNS infection	66 (9.3%)
Cryptococcosis meningitis or meningoencephalitis	27
HIV encephalopathy	14
Tuberculous meningitis	12
CMV meningitis	7
*Toxoplasma gondii* encephalitis	2
Varicella-zoster virus meningitis	2
Neurosyphilis	2
Invasive fungal infection	42 (5.9%)
Cryptococcal infection	32
Talaromyces marneffei infection	6
Aspergillosis infection	4
Cytomegalovirus retinitis	38 (5.3%)
Malignant tumor	34 (4.8%)
Lymphoma	15
Kaposi's sarcoma	9
Others	10
Bacterial bloodstream infection	29 (4.1%)
Non-typhoidal salmonella	15
Others	14
Disseminated *Mycobacterium avium* complex disease	9 (1.3%)
Serum IgM antibody to *Mycoplasma pneumoniae*	44 (6.2%)
Serum antibody to *Legionella pneumophila*	2 (0.3%)
Available positive bacteria from sputum	28 (3.9%)
*Klebsiella pneumoniae*	16
CD4 T counts <200 (/μL)	606 (85.2%)
CRP (>10 mg/L)	511 (71.9%)
Albumin (<30 g/L)	304 (42.8%)
ALT (>50 × U/L)	250 (35.2%)
Hyponatremia	237 (33.3%)
HB (<9 g/L)	87 (12.2%)
Thrombocytopenia	57 (8.0%)
Scr (>104 μmol/L)	13 (1.8%)

*ART, antiretroviral therapy; BMI, body mass index; PJP, pneumocystis jiroveci pneumonia; TB, tuberculosis; CNS, central nervous system; HIV, human immunodeficiency virus; CMV, cytomegalovirus; CRP, C-reactive protein; ALT, glutamic alanine transaminase; HB, hemoglobin; Scr, serum creatinine*.

There were no significant statistical differences in gender, age, BMI <16 kg/m^2^, admission after June 15, 2016, hospitalization days, smoking status, virological failure, elevated ALT and CRP levels, and occurrence of viral hepatitis, hyperlipidemia, PJP, bacterial bloodstream infection between two groups. Compared with the patients who survived, those who died had a higher possibility of being at WHO III–IV stage, having CD4 <200 cells/μL, hyponatremia, thrombocytopenia, HB <9 g/dL, Scr level ≥104 μmol/L, respiratory failure, prevalent tuberculosis, CNS infection, invasive fungal infection, malignancies, and fever duration >7 days before admission, while they had a lesser possibility of undergoing ART prior to admission (Table 2).

**Table 2:** Comparison of the baseline clinical features between survivors and the dead.

**Characteristics**	**Died** **(*n* = 62)**	**Survived** **(*n* = 649)**	***P***
Fever >7 days before admission [*n* (%)]	50 (80.6%)	406 (62.6%)	0.005
ART prior to admission [*n* (%)]	18 (29.0%)	290 (44.7%)	0.017
WHO clinical stage III–IV [*n* (%)]	62 (100%)	470 (72.4%)	0.013
CD4 (/μL) [*n* (%)]			<0.001
<200	62 (100%)	537 (82.7%)	
≥200	0 (0%)	112 (17.3%)	
HB <9 g/dL [(*n* %)]	16 (25.8%)	71 (10.9%)	0.001
Thrombocytopenia [*n* (%)]	10 (16.1%)	47 (7.2%)	0.017
Scr ≥104 μmol/L [(*n* %)]	4 (6.5%)	9 (1.4%)	0.022
Hyponatremia [*n* (%)]	32 (51.6%)	205 (31.6%)	0.020
Respiratory failure [*n* (%)]	23 (37.1%)	124 (19.1%)	0.001
TB [*n* (%)]	37 (59.7%)	245 (37.8%)	0.001
Invasive fungal infection [*n* (%)]	8 (12.9%)	34 (5.2%)	0.018
CNS infection [*n* (%)]	13 (21.0%)	53 (8.2%)	0.001
Malignancies [*n* (%)]	9 (14.5%)	25 (3.9%)	<0.001

*P < 0.05, statistically significant with the use of chi-square test or Fisher's exact. ART, antiretroviral therapy; HB, hemoglobin; Scr, serum creatinine; TB, tuberculosis; CNS, central nervous system*.

### Risk Factors for Mortality

**Figure 1:**
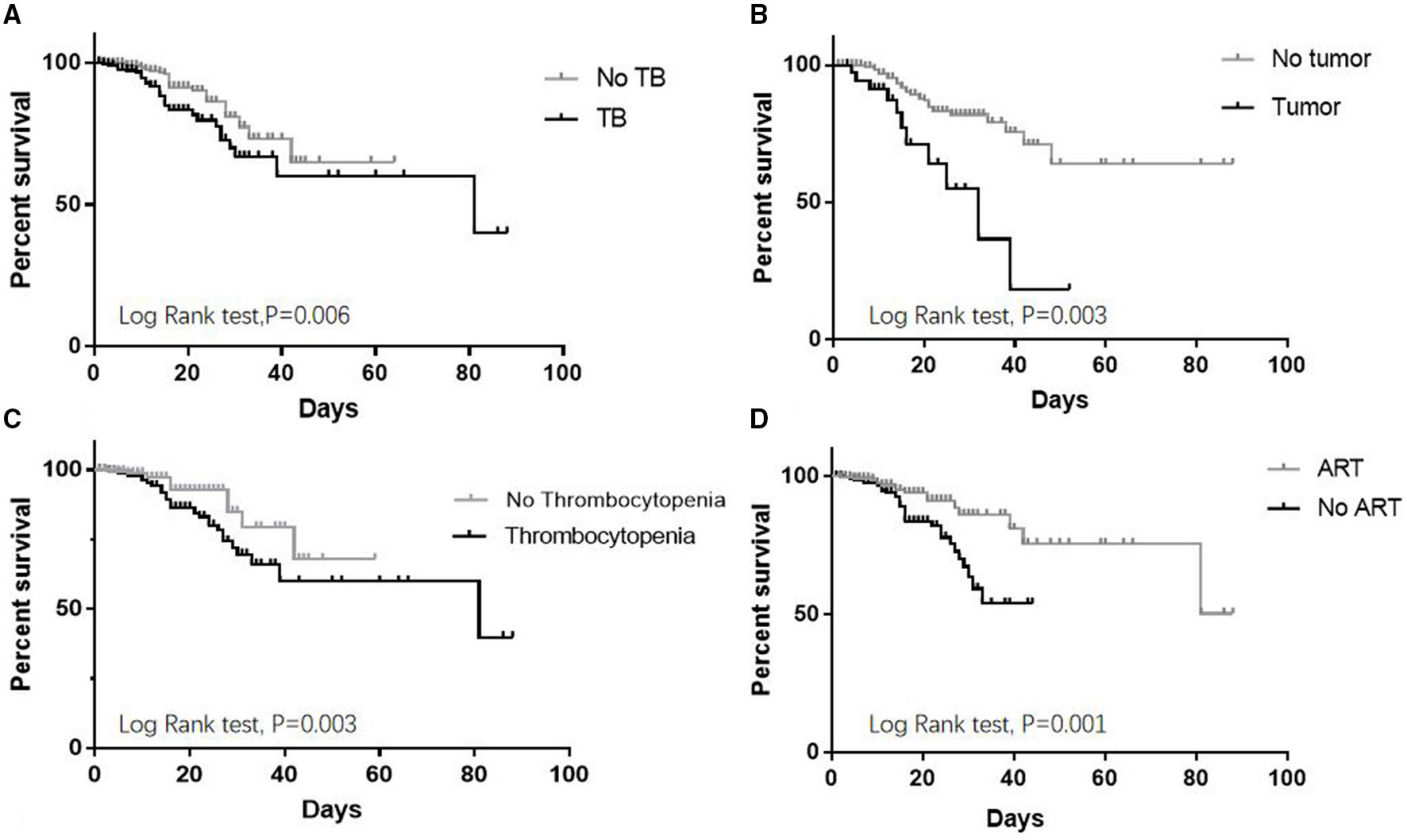
Kaplan–Meier curve for risk factors of mortality in HIV-infected inpatients. **(A)** Prevalent TB increased in-hospital mortality. **(B)** Malignant tumors increased in-hospital mortality. **(C)** Thrombocytopenia increased in-hospital mortality. **(D)** ART before admission decreased in-hospital mortality.

In total, 62 patients died in the hospital, with an in-hospital mortality rate of 8.72%. Approximately, 17.7% (11/62) and 51.6% (32/62) of all deaths occurred within the first week and first 2 weeks of hospitalization, respectively. The causes of death included PJP (21), TB (16), sepsis syndrome (6), malignant tumor (5), cryptococcal meningitis (5), HIV encephalopathy (5), invasive pulmonary aspergillosis infection (3), and hepatic failure (1). Cancer-related deaths accounted for 8.1% of the deaths. Non-AIDS-related deaths occurred in 5 patients (8.1%), including non-AIDS-related malignancies (4) and hepatic failure (1). Data with a *P* < 0.1 in a univariable analysis and age division were entered into the multivariate Cox proportional hazard model including age (*P* > 0.1), admission after June 15, 2016 (*P* = 0.035), presence of thrombocytopenia (*P* < 0.001), HB <9 g/dL (*p* = 0.002), Scr ≥104 μmol/L (*p* = 0.026), being at WHO stage III–IV (*P* = 0.036), CD4 <200 cells/μL (*P* = 0.087), prevalent TB (*P* = 0.006), malignancies (*P* = 0.005), ART status prior to admission (*P* = 0.002), and fever duration >7 days before admission (*P* = 0.026). Finally, analyses using multivariate Cox regression after adjusting for age division and Kaplan–Meier methods showed that prevalent TB (HR: 2.224; 95% CI, 1.322–3.742; *P* = 0.003) (log-rank test, *P* = 0.006), malignancies (HR: 3.933; 95% CI, 1.874–8.256; *P* < 0.001) (log-rank test, *P* = 0.003), and thrombocytopenia (HR: 3.673; 95% CI, 1.814–7.436; *P* < 0.001) (log-rank test, *P* = 0.003) were risk factors for death, while undergoing ART prior to admission (HR: 0.308; 95% CI, 0.169–0.563; *P* < 0.001) (log-rank test, *P* = 0.001) was a protective factor (Figure 1 and Table 3).

**Table 3:** Factors associated with in-hospital mortality among HIV-infected inpatients in Shenyang.

**Factors**	**HR (95%Cl)**	**P1-value**	**Adjust HR(95%Cl)**	**P2-value**
**Age**				
<30	1.000		1.000	
30–49	1.131 (0.600–2.134)	0.703	0.986 (0.517–1.880)	0.965
≥50	0.949 (0.439–2.050)	0.894	0.661 (0.301–1.452)	0.302
**Admission after 20160615**				
Before 20160615	1.000			
After 20160615	0.495 (0.257–0.953)	0.035		
**Thrombocytopenia**				
No	1.000		1.000	
Yes	3.135 (1.782–5.517)	<0.001	3.673 (1.814–7.436)	<0.001
**WHO clinical stage**				
I–II	1.000			
III–IV	2.977 (1.076–8.237)	0.036		
**CD4 <200 (/μL)**				
No	1.000			
Yes	23.993 (0.628–916.609)	0.087		
**Prevalent TB**				
No	1,000		1.000	
Yes	2.081 (1.24–3.492)	0.006	2.224 (1.322–3.742)	0.003
**Malignancies**				
No	1.000		1.000	
Yes	2.765 (1.36–5.623)	0.005	3.933 (1.874–8.256)	<0.001
**ART prior to admission**				
No	1.000		1.000	
Yes	0.401 (0.226–0.71)	0.002	0.308 (0.169–0.563)	<0.001
**Scr ≥104 μmol/L**				
No	1.000			
Yes	3.226 (1.166–8.920)	0.026		
**HB <9 g/dL**				
No	1.000			
Yes	2.533 (1.430–4.487)	0.002		
**Fever >7 d before admission**				
No	1.000			
Yes	2.103 (1.094–4.044)	0.026		

*P1, P-value in a univariable analysis; P2, P-value in the multivariable analysis using Cox regression; TB, tuberculosis; ART, antiretroviral therapy; Scr, serum creatinine; HB, hemoglobin*.

### Trend Analysis by Linear-by-Linear Association Test

**Figure 2:**
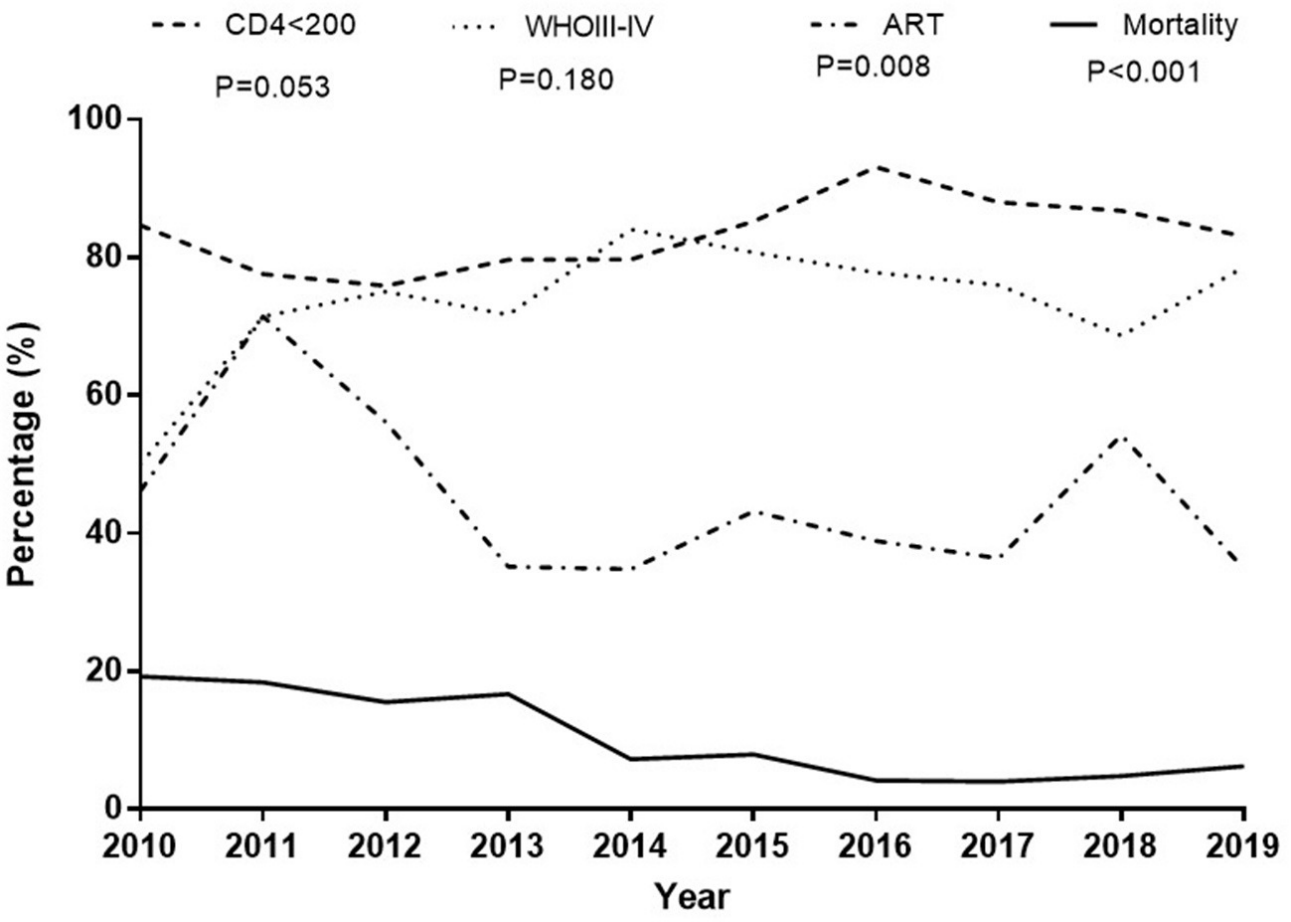
Trend analysis by Linear-by-Linear Association test. The proportion of patients with CD4 counts <200 cells/μL did not change, and the proportion of advanced WHO stages (III–IV) among inpatients did not change. The percentage of patients taking ART before admission was declining; there has been a declining trend in hospital mortality in the past decade.

In the past decade, the trend of in-hospital mortality rates decreased from 19.2% in 2010 to 6.3% in 2019 (linear-by-linear association test, *p* < 0.001), and the percentage of patients who had received ART before admission decreased from 46.2% in 2010 to 34.8% in 2019 (linear-by-linear association test, *p* = 0.008). The prevalence of advanced WHO stages (III–IV) and CD4+T cell counts <200 cells/μL among inpatients did not change (linear-by-linear association test, *p* = 0.180 and *p* = 0.053, respectively) (Figure 2).

## Discussion

Our study, conducted in the general hospital of Northeast China, showed that AIDS-related illnesses remained the leading causes of hospital admission and in-hospital mortality, which was consistent with previous reports (4, 5). PJP and TB remained the top two direct causes of admission and mortality in HIV-infected patients, which is consistent with previous findings (6–8). Patients with respiratory failure can benefit from early admission to critical care (9). Adverse drug event was also reported as an important cause of hospital admission, especially before June 15, 2016, which was mainly due to nevirapine and zidovudine application. As an increasing risk factor of mortality, cancer-related mortality accounted for 8.1% of all deaths in our study, which was lower than that reported in published studies (10, 11), with many patients undergoing long-term ART. Importantly, ART before admission is a protective factor against death.

The rate of non-AIDS-related morbidity as the cause of hospital admission varies with age and geographical area. Most patients in our study were MSM, who might be at a younger age on admission, and this was less associated with underlying medical conditions such as age-related comorbidities. The rate of non-AIDS-related mortality was highest in high-income countries, followed by developing countries, and lowest in sub-Saharan countries (12). The percentage of non-AIDS-related deaths was only 8.1% in this study, which was much lower than that in other studies of long-term follow-up of cohorts undergoing ART which included a large number of patients reaching the age at which malignancies and cardiovascular disease incidence increase (2, 3). In this study, bacterial infection was also a common factor for hospital admission, which is consistent with previous studies (4, 13). The top pathogen of bacterial bloodstream infection is the non-typhoid salmonellae (14). In contrast, bacterial pneumonia in patients with HIV was not associated with higher mortality rate than in the those without HIV (15). Although thrombocytopenia is not a direct cause of death in this study, its severity and incidence were related to the stage of HIV infection, while its incidence markedly decreased during ART (16, 17). A decrease in platelet count is due to peripheral destruction in the early stages of HIV disease, resembling immune thrombocytopenic purpura. In more advanced stages of the disease, low platelet counts are mainly due to injured platelet production and ineffective hematopoiesis (18). Due to the low proportion of ART-experienced patients in this study, drug resistance was not associated with increased mortality rate, which was different from the findings of a study that only enrolled ART-experienced individuals (19). However, the prevalence of virological failure among inpatients was 20.4% in our study, which was slightly lower than that in a study (32%) of HIV drug resistance among hospital inpatients (1), while it was much higher than 7.8% reported in outpatients in a study (20). Therefore, patients with advanced disease require more frequent viral load testing because of poor health status and probable drug-drug interactions.

We observed that three fourth of the patients presented with advanced HIV disease and CD4 counts <200 cells/μL, which did not improve in the past decade. A declining trend in the percentage of patients undergoing ART before admission was because of a decreasing rate of admission due to ART adverse events. It was noted that there was a declining trend in in-hospital mortality in the past decade, which was partly due to an intensified medical care strategy (21–24). We performed regular screening for TB infection, CMV infection, cryptococcal infection, *Toxoplasma gondii* infection, and *Penicillium marneffei* infection in HIV inpatients as well as regular brain, lung, and abdominal computed tomography scans. The HIV specialist team was comparatively fixed, as opposed to the physician's rotation system. Medical specialists, including intensive care unit physicians, ophthalmologists, respiratory endoscopy physicians, neurologists, neurosurgeons, hematologists, microbiologists, medical imaging professionals, and pathologists, joined the MDT for patients diagnosed with AIDS. Discussion, lectures, paper reading, and writing were regularly performed. A prompt diagnosis strategy followed by targeted treatment and early ART could significantly reduce early mortality (25).

Despite universal access to ART guided by an updated strategy of its initiation since June 15, 2016, in China, patients with HIV in Northeast China are still mainly dying of AIDS-related illnesses, an indicator of late diagnosis. Although ART substantially decreases hospitalization rates (25), most patients are unaware of their HIV-infected status before admission. Widespread HIV testing programs, such as home-based testing and self-testing, have been recommended by the WHO (26). Furthermore, HIV testing coverage in all hospital-based departments, especially the emergency department, should be recommended (27). Preventive therapy with co-trimoxazole and isoniazid should be reinforced to prevent PJP, bacterial pneumonia, and TB (26). Finally, low-cost interventions such as training of HIV care clinic staff may be the key to reducing early in-hospital mortality. Our study showed significant for decreasing the mortality rates in mortality rates among inpatients with advanced HIV disease in Northeast China.

Our study has some limitations: first, the sample size was small; second, we only enrolled hospitalized patients in the Department of Infectious Disease in our hospital because there was a lack of intensified medical care strategy and professional assessment of HIV inpatients in other departments; third, in this retrospective study, the CD4+T cell counts among inpatients were recorded according to the patients' statement; therefore, we did not adjust for the levels of CD4+T cell counts.

In summary, AIDS-related illnesses remain a prominent problem for in-hospital patients. We must highlight the continuing contribution of TB and malignancies to in-hospital mortality in the people diagnosed with HIV. Further evaluation of the impact of HIV testing coverage in all hospitalized patients and the intensified medical care strategy for decreasing in-hospital mortality is crucial. HIV diagnosis and ART should be carried out as early as possible, as this can ultimately decrease both AIDS mortality and non-AIDS-related mortality.

## Data Availability Statement

The raw data supporting the conclusions of this article will be made available by the authors, without undue reservation.

## Ethics Statement

The studies involving human participants were reviewed and approved by the Clinical Research Ethics Committee of China Medical University. Written informed consent to participate in this study was provided by the participants' legal guardian/next of kin.

## Author Contributions

CL was responsible for data collection, statistical analysis, and article writing. YZ, YWa, SL, WW, XL, CS, and PL were participated in the clinical diagnosis and treatment. YWe designed the article and took part in writing and revising. Q-HH designed the article and was responsible the statistical analysis. All authors contributed to the article and approved the submitted version.

## Funding

This work was supported by the Double First-Class University and discipline construction funds of China Medical University (3110119068 to YWe) and the Fund of National Natural Science (82073620 to Q-HH). The funders had no role in the study design, data collection, and analysis.

## Conflict of Interest

The authors declare that the research was conducted in the absence of any commercial or financial relationships that could be construed as a potential conflict of interest.

## Publisher's Note

All claims expressed in this article are solely those of the authors and do not necessarily represent those of their affiliated organizations, or those of the publisher, the editors and the reviewers. Any product that may be evaluated in this article, or claim that may be made by its manufacturer, is not guaranteed or endorsed by the publisher.

## Abbreviations

AIDS: acquired immune deficiency syndrome
HIV: human immunodeficiency virus
ART: antiretroviral therapy
MDT: multidisciplinary team
BMI: body mass index
WHO: World Health Organization
HB: hemoglobin
PLT: platelet
ALT: glutamic alanine transaminase
ALB: albumin
Scr: serum creatinine
CRP: C-reactive protein
TB: tuberculosis
PJP: pneumocystis *jiroveci* pneumonia
CNS: central nervous system
HR: hazard ratio
CI: credibility interval
MSM: men who have sex with men
CMV: cytomegalovirus.
